# Inequality of obstetric and gynaecological workforce distribution in China

**DOI:** 10.1186/s12939-017-0716-6

**Published:** 2018-01-05

**Authors:** Zhenghong Ren, Peige Song, Xinlei Chang, Jiawen Wang, Lin An

**Affiliations:** 1 Department of Maternal and Child Health, School of Public Health, Peking University, 38 Xueyuan Road, Beijing, 100191 China; 2 Centre for Population Health Sciences, University of Edinburgh, Edinburgh, EH8 9AG UK; 3 Institute of Medical Humanities, Peking University, Beijing, 100191 China

**Keywords:** Obstetric and gynaecological workforce, Health human resources, Inequality, China

## Abstract

**Background:**

Women’s health is defined as a continuum throughout their whole lives. In China, women receive life-round preventative and curative health care from the health system, although the universal access to reproductive health has already been basically achieved in China, the situation of women’s access to curative health care is still unknown.

**Methods:**

Data from the national maternal and child health human resource investigation were analysed. Lorenz curves, Gini coefficients, and Theil L indexes were drawn and calculated to reflect the inequality. Demographically, we found that the Obstetric and gynaecological (OB/GYN) workforce was the least equitable regarding the distribution of live births.

**Results:**

Demographically, we found that the OB/GYN workforce was the least equitable regarding the distribution of live births. The geographic distribution of the OB/GYN workforce was found to be severely inequitable, especially in the West region. Most of the inequality was found to come from inner-regions.

**Conclusion:**

For the first time, the distribution inequality of OB/GYN workforce in China was analysed. The findings in this study can be adopted in making national or regional OB/GYN workforce allocation policies, but further studies are still needed to reveal the detailed sources of inequality and to provide evidence for local policy-making.

**Electronic supplementary material:**

The online version of this article (10.1186/s12939-017-0716-6) contains supplementary material, which is available to authorized users.

## Background

Women’s health involves women’s emotional, social and physical well-being [1], and is defined as a continuum throughout their whole lives [2, 3]. Due to the gender-specific intersection of biology and related sociocultural factors, the top morbidities for women and girls are different from men and boys [4, 5]. In the past few decades, the importance of women’s health has been increasingly recognised across the whole world [6], especially after one and a half decades’ global collaboration on the Millennium Development Goals (MDGs), remarkable progress has been made in reducing maternal mortality and improving maternal health [7]. In the MDGs era, as the biggest developing country, China has made remarkable progress in improving maternal health, and successfully achieved the MDG 5 by reducing its maternal mortality rate by three quarters from 1990 to 2015 (MDG 5.A) and by basically achieving universal access to reproductive health services (MDG 5.B) [8].

In the new era of the Sustainable Development Goals (SDGs), the SDG 3 calls for continuous global and domestic efforts to achieve the universal health coverage and to make sure that every person can have equitable access to affordable, accountable, appropriate health services of assured quality [9, 10]. However, when compared with men, women in most of the countries are still suffering more restricted access to health resources [3, 11], this is also true for Chinese women [12]. In China, women receive life-round preventative and curative health care from the health system, the universal access to reproductive health has already been basically achieved in China when being measured by the free basic technical services of family planning and systematic maternal management coverage [8]. However, the situation of women’s access to curative health care is still unknown.

Reproductive health is central to women’s health [3]. Obstetrics and Gynaecology (OB/GYN) are the basic medical and surgical specialities that deal with the female reproductive organs during women’s pregnant/non-pregnant states [13]. Obstetricians, gynaecologists, nurses and midwives are the professional frontline curative service providers for women’s reproductive health. Globally and nationally, establishing the Obstetric and Gynaecological (OB/GYN) workforce allocation standards is important but notoriously challenging [14]. In China, there is still no related national or local regulations. The distribution of OB/GYN resources, such as the number of delivery beds and the number of OB/GYN specialists varies hugely across the country [15]. In such contexts, universal health coverage for women’s health has become a distant objective, especially in rural areas [16].

In the National Maternal and Child Health (MCH) Human Resources Investigation [17, 18], the MCH professionals in China were sufficient for their workload in 2010 at the national level [18]. However, concerns have been voiced on the debate regarding the inner-country misdistribution of OB/GYN workforce in China, especially for the urban-rural disparity [14]. For women’s health, the deficiency of OB/GYN workforce is significant for resource-poor settings and thus can increase the risk of poor health outcomes of women living in less-developed areas where access to adequate women’s health care is insufficient [19]. With the absence of national or local standards of OB/GYN workforce allocation standards, efforts should be made to reveal the distribution situation of OB/GYN workforce in China to inform policy-making. In this study, our analysis shed lights on the distribution inequality of OB/GYN workforce in China by using the data from the national investigation of maternal and child health human resource.

## Methods

### Sampling methods

**Fig. 1:**
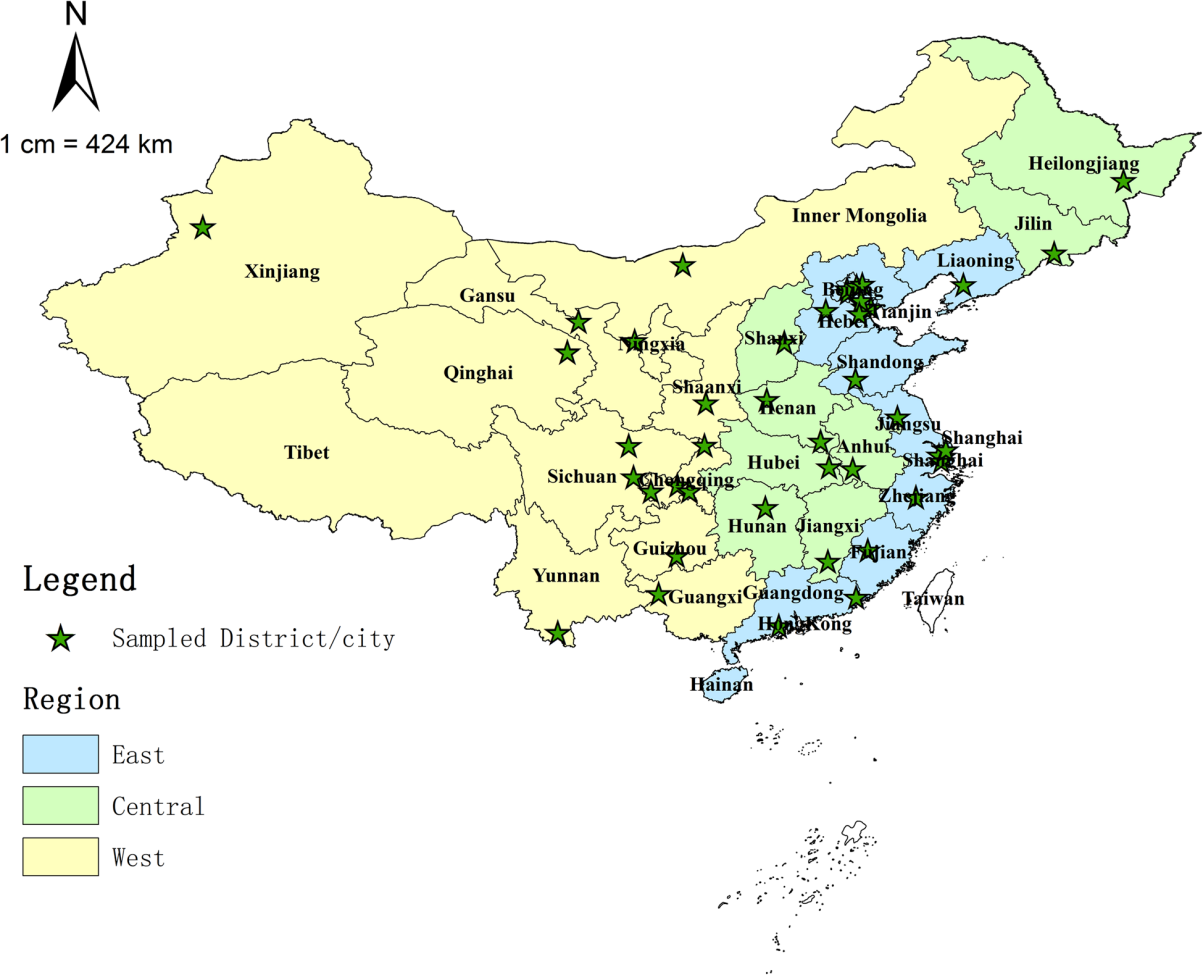
Map of China showing the East, Central, and West regions and sampled districts/cities. Notes: the three regions (East, Central, and West) were categorised according to the China Health and Family Planning Yearbook [31]

The national investigation of maternal and child health human resource was a national institution-based sampling survey, it is initiated and supported by the National Health and Family Planning Commission of the People’s Republic of China (the former Chinese Ministry of Health until 2013) [20, 21]. The detailed information about the investigation methods has been published and described elsewhere [17, 18, 22]. Firstly, among the 22 provinces and 5 autonomous regions in China, 28 districts/cities were selected as samples from the 332 municipality Prefectural districts/cities using random clustering sampling. For the four municipalities (Beijing, Shanghai, Tianjin and Chongqing), two urban districts and two rural counties were sampled from every municipality respectively. Finally, 44 districts/cities were included as sampled areas in this survey, all of which came from province-level administrative divisions in China except for Tibet Autonomous Region, Hainan province (there are only a few cities), Hong Kong Special Administrative Region, Macau Special Administrative Region and Taiwan Province. The geographic distribution of the sampled areas is shown in Fig. 1.

### Data collection

A structured questionnaire was developed and improved after several rounds of expert consultation. Before the formal investigation, the questionnaire was piloted in September 2011. From November 2011 to February 2012, the questionnaire was sent to all the medical and healthcare institutions providing maternal and child health services within the sampled districts/cities. All data on population size, population structure, and geographic area, etc., were obtained from the local governments, and the information about the number of health workers was obtained from the institutions. When counting the number of workers, according to the expert consultation, a weight of 0.5 was assigned to workers who were not working full-time to avoid overestimation of the total workforce. The total number of workers in every institution within the sampled district/city was then added together to reveal the total size of the workforce in the sampled district/city. All data was then anonymised, sent back by post mail, and we were granted permission to use and analyse the data by the participating districts/cities and institutions. In this study, our targeted professionals were obstetricians, gynaecologists, nurses and midwives who worked in the OB/GYN departments. The categorization of the workforce was based on the professionals’ holding certificates.

### Inequality assessment

In this study, Lorenz curve, Gini coefficient and Theil L index were chosen as the indicators for assessing the inequality of the health resources distribution [22, 23]. The Lorenz curve and Gini coefficient are widely adopted in evaluating the inequality in the area of economics as well as the public health research area [24, 25]. In the Lorenz curve, the x-axis represents the cumulative share of population or geography, the y-axis indicates the cumulative share of the OB/GYN workforce, and the ideal equality distribution is a diagonal line, the larger the Lorenz curve distance from the ideal equality line, the greater the inequality [26]. The Gini coefficient was calculated as the ratio of the area between the Lorenz curve and the ideal equality line. The larger the Gini coefficient, the greater the inequality. The levels of inequality were defined as: a Gini coefficient < 0.2 indicates absolute equality, 0.2–0.3 relative equality, 0.3–0.4 proper inequality, 0.4–0.5 large inequality, and above 0.5 represents severe inequality [27]. The formula adopted in this study for calculating the Gini coefficient is:

1 $$ \mathrm{G}=1-\sum \limits_{i=0}^{k-1}\left({CY}_{i+1}+{CY}_i\right)\left({CX}_{i+1}-{CX}_i\right) $$

where *G* is the Gini coefficient; *CY*_*i*_ is the cumulative proportion of the OB/GYN workforce (obstetricians, gynaecologists, nurses and midwives) in the *ith* district/city; *CX*_*i*_ is the cumulative proportion of the demographic/geographic variable (number of targeted population or geographic area) in the *ith*district/city; and *k* is the total number of the districts/cities [28].

The Theil L index is also a widely adopted indicator to detect the inequality, when comparing with the Lorenz curve and the Gini coefficient, it has the advantage of decomposition, which means decomposing the total national inequality to inner-regional difference and inter-regional difference [28, 29]. The larger the Theil L index, the greater the inequality. However, the Theil L index is a relative indicator, so there is no universal assessment standard of inequality levels [30]. The formula adopted in this study for calculating the Theil L index is:

2 $$ L={\sum}_i\left(\raisebox{1ex}{${X}_i$}\!\left/ \!\raisebox{-1ex}{$X$}\right.\right)\log \left[\left(\raisebox{1ex}{${X}_i$}\!\left/ \!\raisebox{-1ex}{$X$}\right.\right)/\left(\raisebox{1ex}{${Y}_i$}\!\left/ \!\raisebox{-1ex}{$Y$}\right.\right)\right] $$

where L is the Theil L index; *Y*_*i*_ is the proportion of the OB/GYN workforce (obstetricians, gynaecologists, nurses and midwives) in the *ith* district/city; and *X*_*i*_ is the proportion of the demographic/geographic variable (number of targeted population or geographic area) in the *ith* district/city [29].

### Statistical analysis

In the investigation, it was difficult to clearly classify obstetricians and gynaecologists, especially in the small-scale health institutions. In the analysis, obstetricians and gynaecologists were gathered together as OB/GYN doctors, then the numbers of OB/GYN doctors, OB/GYN nurses, midwives, and total OB/GYN workforce per 10,000 population, per 10,000 women ≥15 years, per 10,000 women of reproductive age (15–49 years), per 1000 live births were calculated to represent the OB/GYN workforce’s demographic distribution inequality; the numbers of OB/GYN doctors, OB/GYN nurses, midwives, and total OB/GYN workforce per square kilometre were calculated to access the OB/GYN workforce’s geographic distribution inequality.

Then the sampled districts/cities were categorised into three regions: East, Central, and West, where the East region is the most developed area, the Central region is less developed than the East, and the West is the least developed. According to the National Health and Family Planning Commission of the People’s Republic of China, the East region includes 11 provinces: Beijing, Tianjin, Hebei, Liaoning, Shanghai, Jiangsu, Zhejiang, Fujian, Shandong, Guangdong and Hainan; the Central region includes eight provinces: Shanxi, Jilin, Heilongjiang, Anhui, Jiangxi, Henan, Hubei and Hunan; and the West region includes 12 provinces: Inner Mongolia, Guangxi, Shaanxi, Gansu, Qinghai, Ningxia, Xinjiang, Sichuan, Chongqing, Guizhou, Yunnan and Tibet (Fig. 1) [31].

Finally, the corresponding Lorenz curves were drawn, the Gini coefficients and Theil L indexes were calculated for the above indicators to assess the inequality at the national and regional (East, Central, and West) levels, and the Theil L indexes were then decomposed to assess the share of inner-regional and inter-regional inequality.

The geographic distribution map of the sampled districts/cities was drawn using ArcGIS 10.1 (Environmental Systems Resource Institute, Redlands, CA, USA), all analyses were conducted in SPSS 13.0 (SPSS Inc., Chicago, IL, USA), and the Lorenz curves and proportional bar charts were drawn using Microsoft Excel 2013 (Microsoft Corporation, Redmond, WA, USA).

## Results

There were 60,207 OB/GYN workers in the 44 investigated districts/cities, among them, 26,776 (44.5%) were OB/GYN doctors, 23,465 (39.0%) were OB/GYN nurses, and 9966 (16.6%) were midwives. Demographically, there were 4.52 OB/GYN workers per 10,000 population, 10.94 per 10,000 women ≥15 years, 15.98 per 10,000 women of reproductive age, and 43.51 per 1000 live births; Geographically, the average number of OB/GYN workers per square kilometre was 0.080. The detailed demographic and geographic distributions of OB/GYN workers in the 44 sampled districts/cities are shown in Additional file 1: Tables S1 and S2.

**Fig. 2:**
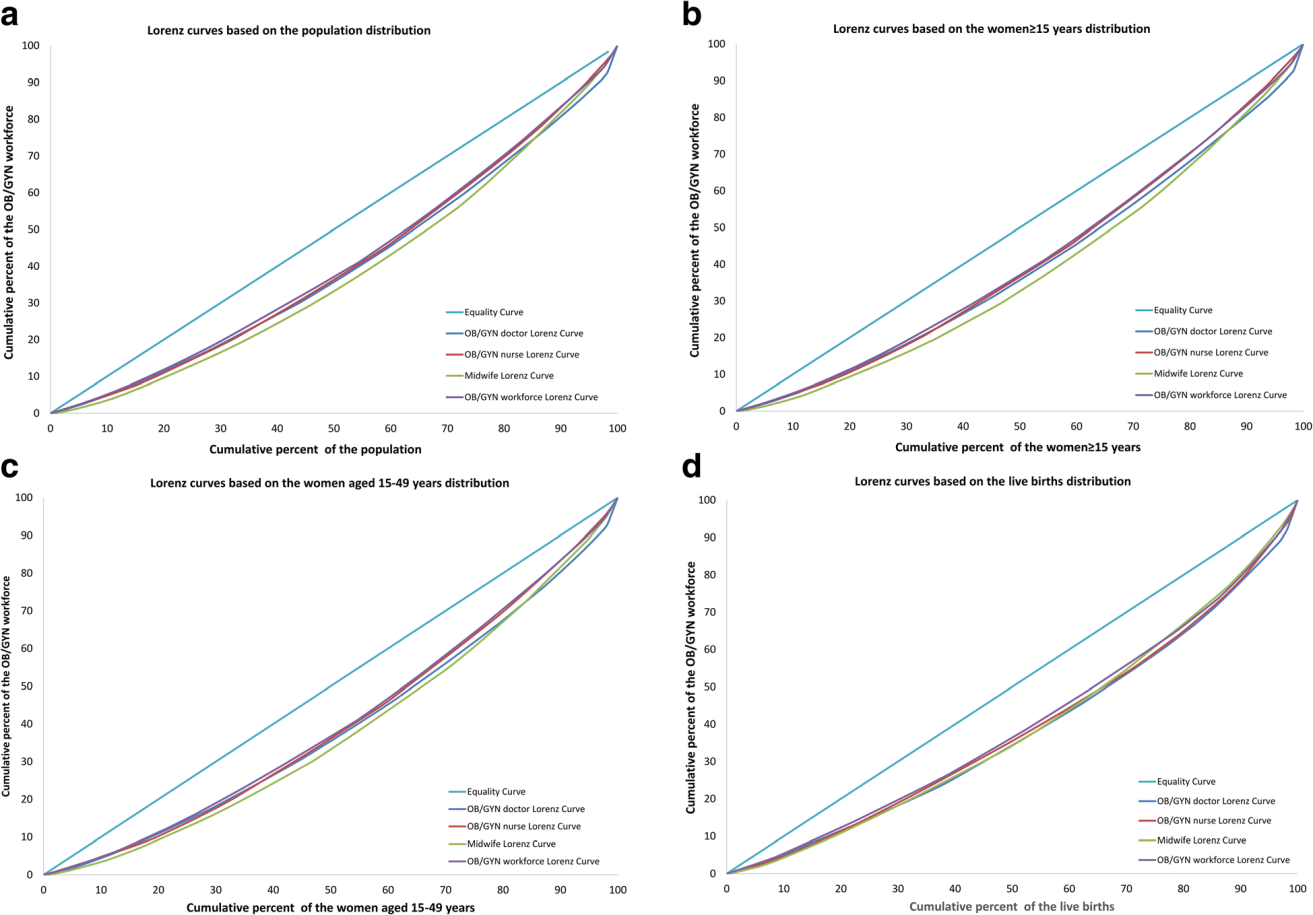
Lorenz curves for the demographic distributions of different indicators. **a** Based on the population distribution; **b** Based on the women ≥15 years distribution; **c** Based on the of reproductive age distribution; **d** Based on the live births distribution

**Fig. 3:**
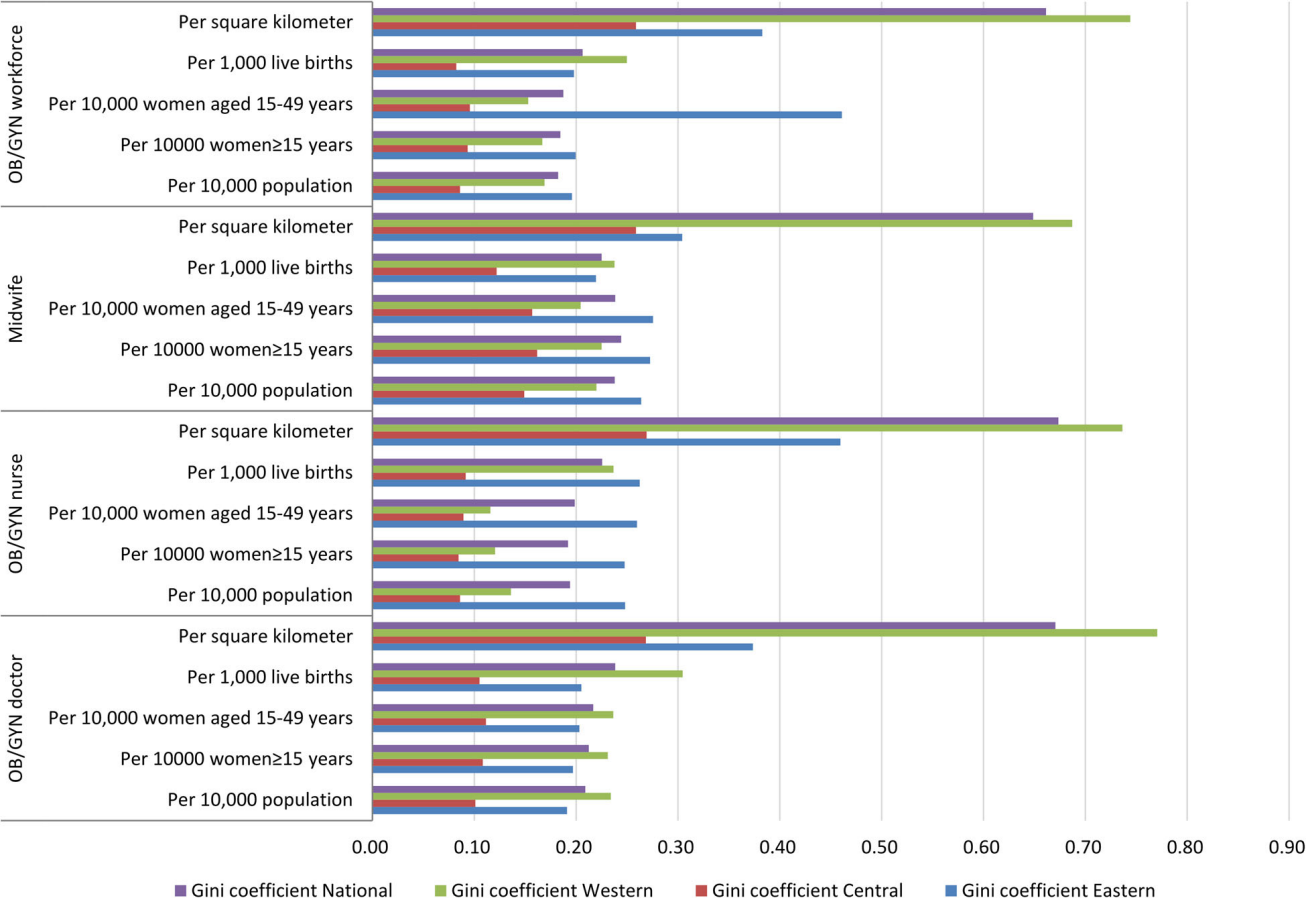
Comparison of Gini coefficients of different indicators at both national and regional levels

**Fig. 4:**
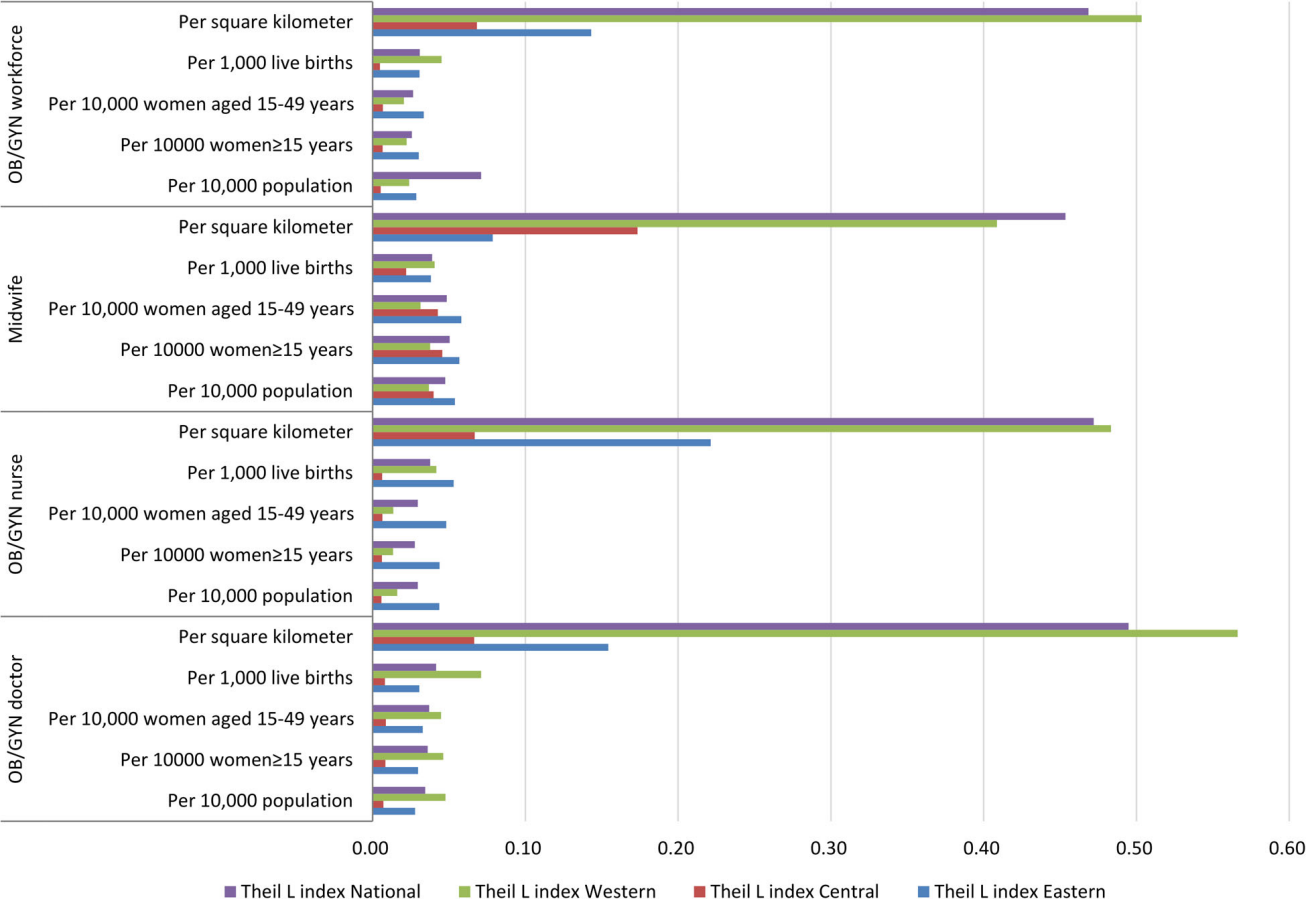
Comparison of Theil L indexes of different indicators at both national and regional levels

**Fig. 5:**
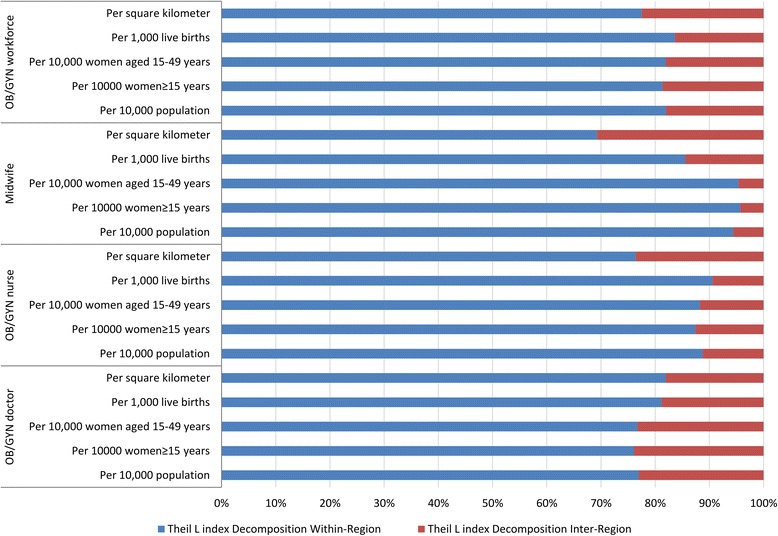
The share of inequality sources by Theil L decomposition

The demographic distribution inequality of OB/GYN workforce measured by the Lorenz curves is shown in Fig. 2, and the corresponding Gini coefficients and Theil L indexes are listed in Table 1. In 2010, the Gini coefficient of the total OB/GYN workforce per 10,000 population in China was 0.182, which indicated an absolute equality, the corresponding comparisons of the Gini coefficients and the Theil L indexes at both national and regional levels are shown in Figs. 3 and 4. Among all the three regions, the East was the most inequitable with the highest Gini coefficient of 0.196 and the highest Theil index of 0.029, but still at the level of absolute equality. The inequality sources are shown in Fig. 5, according to the decomposition of the Theil L, the majority of the inequality (81.98%) of total OB/GYN workforce per 10,000 population came from the inner-regions. When dividing the OB/GYN workforce into sub-groups of OB/GYN doctors, OB/GYN nurses and midwives, the distribution of midwives per 10,000 population was the least equitable comparing with the distributions of OB/GYN doctors and OB/GYN nurses, according to the Gini coefficient standard, the distribution of midwives per 10,000 population was at the level of relative equality (Gini: 0.238), the distributions of OB/GYN doctors per 10,000 population was also at the level of relative equality with a Gini coefficient of 0.209, whereas the distribution of OB/GYN nurses per 10,000 population indicated an absolute equality (Gini: 0.194). Similar to the distribution of the total OB/GYN workforce, the East region was the least equitable among all the three regions regarding the distributions of OB/GYN nurses and midwives per 10,000 population (Gini: 0.248 and 0.264, Theil L: 0.044 and 0.054); the West region was the least equitable regarding the distribution of the OB/GYN doctors per 10,000 population. The main sources of the inequality of the three sub-groups of OB/GYN workforce (OB/GYN doctors, OB/GYN nurses and nurses) were all inner-regional, which accounted for 76.97%, 88.76% and 94.39% of the total inequalities respectively.

**Table 1:** Gini coefficients and Theil L indexes of different indicators in the three regions

Workforce	Variable	Gini coefficient				Theil L index					
		East	Central	West	National	East	Central	West	National	Inner-Region (%)	Inter-Region (%)
OB/GYN doctor	/10,000 population	0.191	0.101	0.234	0.209	0.028	0.007	0.048	0.034	0.026(76.97)	0.008(23.03)
	/10,000 women ≥ 15 years	0.197	0.108	0.231	0.212	0.030	0.008	0.046	0.036	0.027(76.07)	0.009(23.93)
	/10,000 women aged 15–49 years	0.203	0.111	0.236	0.217	0.033	0.009	0.045	0.037	0.028(76.74)	0.009(23.26)
	/1000 live births	0.205	0.105	0.305	0.238	0.031	0.008	0.071	0.042	0.034(81.16)	0.008(18.84)
	/square kilometre	0.374	0.269	0.770	0.670	0.154	0.066	0.566	0.495	0.405(81.95)	0.089(18.05)
OB/GYN nurse	/10,000 population	0.248	0.086	0.136	0.194	0.044	0.006	0.016	0.030	0.024(88.76)	0.003(11.24)
	/10,000 women ≥ 15 years	0.248	0.085	0.120	0.192	0.044	0.006	0.013	0.028	0.024(87.41)	0.003(12.59)
	/10,000 women aged 15–49 years	0.260	0.089	0.116	0.199	0.048	0.006	0.014	0.030	0.026(88.25)	0.003(11.75)
	/1000 live births	0.262	0.092	0.237	0.226	0.053	0.006	0.042	0.038	0.034(90.60)	0.004(9.40)
	/square kilometre	0.460	0.269	0.736	0.673	0.221	0.067	0.483	0.472	0.361(76.39)	0.111(23.61)
Midwife	/10,000 population	0.264	0.149	0.220	0.238	0.054	0.040	0.037	0.048	0.045(94.39)	0.003(5.61)
	/10,000 women ≥ 15 years	0.273	0.162	0.225	0.244	0.057	0.046	0.038	0.050	0.048(95.73)	0.002(4.27)
	/10,000 women aged 15–49 years	0.276	0.157	0.205	0.238	0.058	0.043	0.031	0.049	0.046(95.37)	0.002(4.63)
	/1000 live births	0.220	0.122	0.238	0.225	0.038	0.022	0.041	0.039	0.033(85.57)	0.006(14.43)
	/square kilometre	0.304	0.259	0.687	0.649	0.079	0.173	0.409	0.454	0.314(69.28)	0.139(30.72)
OB/GYN workforce	/10,000 population	0.196	0.086	0.169	0.182	0.029	0.005	0.024	0.071	0.020(81.98)	0.004(18.02)
	/10,000 women ≥ 15 years	0.200	0.094	0.167	0.185	0.030	0.007	0.022	0.026	0.021(81.37)	0.005(18.63)
	/10,000 women aged 15–49 years	0.461	0.096	0.153	0.187	0.033	0.007	0.021	0.026	0.022(81.97)	0.005(18.03)
	/1000 live births	0.198	0.082	0.250	0.206	0.031	0.005	0.045	0.031	0.026(83.64)	0.005(16.36)
	/square kilometre	0.383	0.259	0.744	0.661	0.143	0.068	0.503	0.469	0.363(77.53)	0.105(22.47)

Note: The three regions (East, Central and West) were categorised according to the China Health and Family Planning Yearbook [31]

The inequality of OB/GYN workforce distribution per 10,000 women (women ≥15 years and women of reproductive age) measured by the Lorenz curve is presented in Fig. 2, and the corresponding Gini coefficients and Theil L indexes are shown in Table 1. The inequalities of total OB/GYN workforce per 10,000 women ≥15 years and per 10,000 women of reproductive age were similar, both indicating absolute equalities (Gini: 0.185 and 0.187 respectively). Among all the three regions, the East was the least equitable and the Central was the most equitable regarding both the distributions of OB/GYN workforce per 10,000 women ≥15 years and per 10,000 women of reproductive age, the inequality of OB/GYN workforce per 10,000 women ≥15 years was at the level of relative equality (Gini: 0.200) in the East region, whereas the inequality of OB/GYN workforce per 10,000 women of reproductive age was at the level of large inequality (Gini: 0.461). According to the Theil L decomposition, the main sources of inequalities of OB/GYN workforce per 10,000 women ≥15 years and per 10,000 women of reproductive age were both inner-regional, accounting for 81.37% and 81.97% of the total inequalities respectively. Regarding different sub-groups of OB/GYN workforce, the Gini coefficients of OB/GYN nurses per 10,000 women ≥15 years and per 10,000 women of reproductive age both indicated absolute equalities (Gini: 0.192 and 0.199 respectively) at the national level, among all the three regions, the distributions of OB/GYN nurses per 10,000 women ≥15 years and per 10,000 women were both least equitable in the East region and reached the level of relative equality (Gini: 0.248 and 0.260). Similarly, the distributions of midwives per 10,000 women ≥15 years and per 10,000 women of reproductive age were both least equitable in the East region, and reached the level of relative equality at the national level; However, the distributions of OB/GYN doctors per 10,000 women ≥15 years and per 10,000 women of reproductive age were both least equitable in the West region.

The number of live births was adopted as the proxy of the women with real maternal care requirements. At the national level, the distribution of OB/GYN workforce per 1000 live births was relatively equitable with a Gini coefficient of 0.206. The West was the least equitable (Gini: 0.250) and the Central was the most equitable (Gini: 0.082), according to the Theil L decomposition, 83.64% of the inequity came from inner-regions. This is a similar situation for the distributions of OB/GYN doctors and midwives per 1000 live births, where the levels of equality both reached relative equality (Gini: 0.238 and 0.225), among all the three regions, the West was also the most inequitable regarding the distribution of OB/GYN doctors and midwives per 1000 live births (Gini: 0.305 and 0.238). The distribution of OB/GYN nurses per 1000 live births also represented a relative equality (Gini: 0.226), but was most inequitable in the West region (Gini: 0.238). Inner-regional inequality was still the main source of total inequality regarding all the distributions of all the sub-groups of OB/GYN workforce per 1000 live births.

**Fig. 6:**
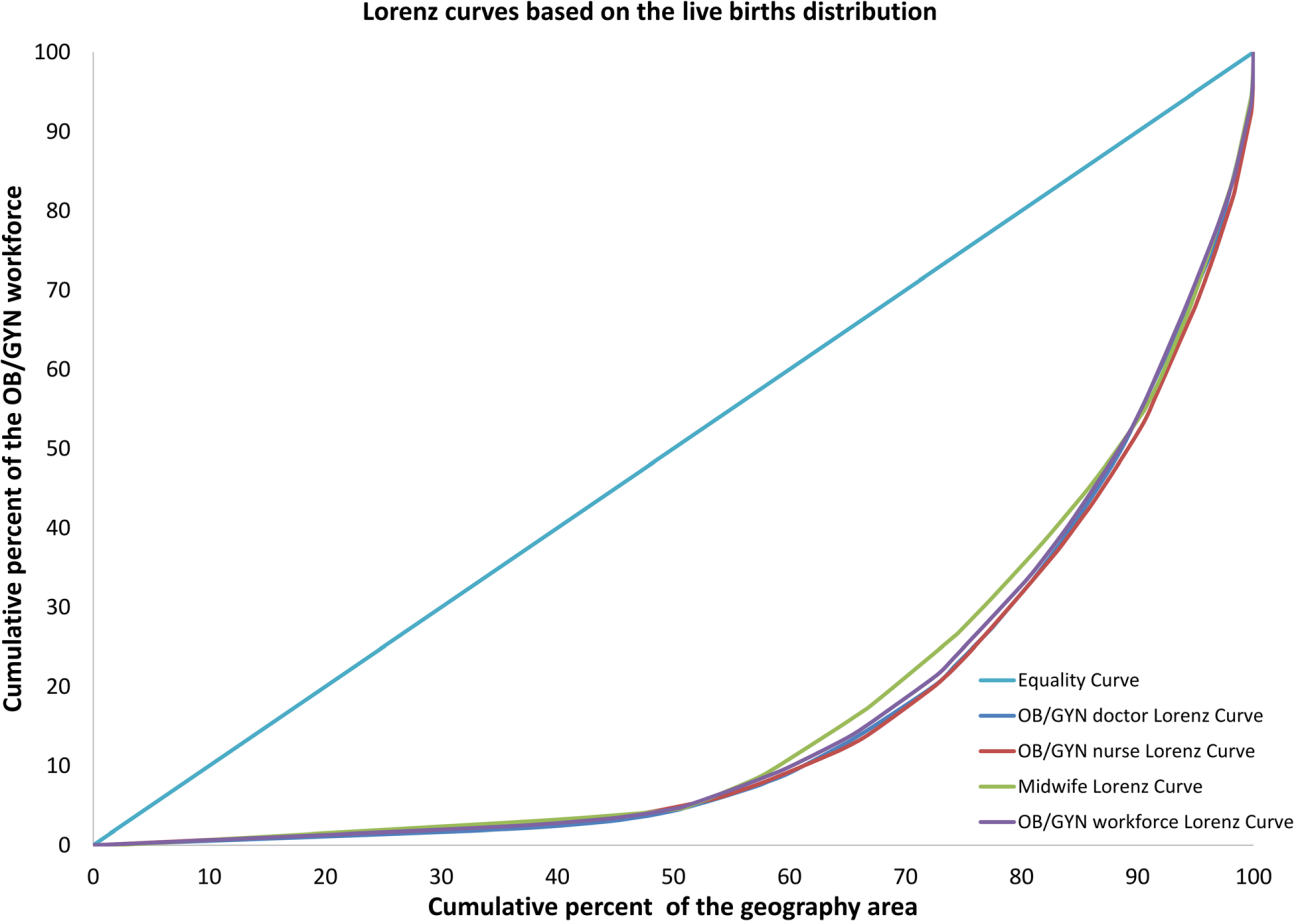
Lorenz curves for the geographic distributions of different indicators

The Lorenz curves based on the geographic distribution of OB/GYN workforce are shown in Fig. 6. At the national level, the distribution of OB/GYN workforce per square kilometre revealed a severe inequality (Gini: 0.661), and most of the inequality came from inner-regions (77.53%). Among all the three regions, the most inequitable region was the West and the least inequitable was the Central, this was also true regarding the distributions of all the sub-groups of OB/GYN workforce. The distributions of OB/GYN doctors, OB/GYN nurses and midwives were all at the level of severe inequality nationally (all Gini coefficients >0.600).

## Discussion

This study is the second paper analysing the inequality of health human resources distribution in China by using the 2010 national maternal and child health human resource investigation. The first paper examined the inequality of paediatric workforce distribution in China [22]. In this study, we assessed the inequality of OB/GYN workforce distribution, to the best of our knowledge, this is the first study with attempts to reveal the distribution of OB/GYN professional workforce at the national and regional level in China.

In this study, the demographic- and geographic-related inequalities of OB/GYN workforce were analysed respectively. Demographically, the distribution of the OB/GYN workforce was least equitable regarding the distribution of live births, the adoption of the number of live births as the proxy of women with real maternal care requirements, i.e., women who gave births in 2010, can partly represent the capacity of women with maternal care requirements when the number of women with real maternal care requirements was not available. Comparing with the distribution of life-round women (≥15 years) and reproductive-aged women (15–49 years), the inequality of OB/GYN workforce distribution of women with maternal care requirements (per 1000 live births) was severer, this may require additional attention on the allocation of maternal professionals. Similar to the study of the inequality of paediatric workforce distribution in China [22], this study also highlighted very severe geographic distribution inequality, especially in the West region, this may not be a huge problem because the West region was less-populous when comparing with the other two regions, but this result still suggests that women in the West region may need to travel a longer distance to receive OB/GYN care services, and this may even cause bad consequences if the distance and transport become a major obstacle of care-seeking behaviours [32].

The levels of inequality varied among different sub-groups of OB/GYN workforce, regarding the demographic distribution (except live births), the level of inequality of midwives was the highest, this may reflect the shortage of midwives, especially in the East region, however, regarding the distribution of live births, the inequality was the highest in the West region, more attention should be given in this maldistribution of midwives because skilled birth attendance is essential for safe delivery, maternal and newborn lives [32, 33]. However, the comparison of inequality of OB/GYN doctors and OB/GYN nurses was not the in line with the results of paediatricians and paediatric nurses, in this study, the OB/GYN doctor distribution was found to be more inequitable than OB/GYN nurse distribution, this may indicate that the OB/GYN doctors were in shortage than OB/GYN nurses. Generally, nurses are regarded as being more flexible in career planning than doctors [22, 34], the contrary results in this study call for further in-depth studies to explore possible reasons.

Similar to the results of paediatric workforce study [22], the inequality of OB/GYN workforce mainly came from inner-regions in this study. The Central region was found to be more equitable than the East and the West regarding both the demographic and the geographic distributions of OB/GYN workforce and for different subgroups of OB/GYN workforce. Although the East region was the most developed area in China [31, 35], the advance of socioeconomic development didn’t bring additional health equality bonus. For the least developed West region, the inequalities of OB/GYN workforce regarding the distributions of total population, women ≥15 years and women of reproductive age were better than that in the East. The possible reason may be the large-scale labour migration into richer areas [35]. However, for women with real maternal requirements, who tend to delivery at hometown [36], their corresponding equality of OB/GYN workforce distribution was severest in the West region, this might be associated with the low economic development in this region.

Despite the advantage of providing both demographic and geographic distribution inequalities for the OB/GYN workforce in China, this study still has several limitations. Firstly, this investigation adopted a national sampling method, we can only assess the inequality at the national and regional levels, no detailed inequality for specific provinces can be captured, neither for rural and urban areas. In addition, we only assessed the sources of inequality at the regional level and found that most of the inequality came from the inner-regions, however, the restriction of exploring more detailed inequality at provincial levels also limited our ability to explore detailed sources of inequality, as well as the reasons for inner-regional inequalities. Secondly, in this study, the classification of OB/GYN workforce was based on the workers’ holding certificates, not based on their real daily work contents, this may lead to misclassification, especially when the workers’ affiliations were small-scale. In addition, although efforts were made to explore the inequality among different OB/GYN sub-groups, the sub-group of OB/GYN doctors actually includes two different groups of professionals, i.e., obstetricians, gynaecologists, this may limit the comparison with other similar studies. Thirdly, different surrogates of demographic distribution were proposed in this study, such as the total population, the life-round women, women of reproductive age and women with real maternal requirements, those indicators are of merits in representing different aspects of OB/GYN workforce distributions, however, the number of live births cannot fully reflect the capacity of women with real maternal care requirements because pregnant women, women who had abortions or stillbirths may also use the maternal care services provided by the OB/GYN departments.

## Conclusions

For the first time, the distribution inequality of OB/GYN workforce in China was analysed. Different types of inequalities still exist across the whole nation of China in 2010. The findings in this study can be adopted in making national or regional OB/GYN workforce allocation policies, but further studies are still needed to reveal the detailed sources of inequality and to provide evidence for local policy-making.

## Additional file

Additional file 1: **Table S1.** The demographic distribution of OB/GYN workforce in the sampled districts/cities; **Table S2.** The geographic distribution of OB/GYN workforce in the sampled districts/cities. (DOCX 27 kb)

## Abbreviations

MCH: Maternal and child health
MDG: Millennium development goal
OB/GYN: Obstetric and Gynaecological/Obstetrics and Gynaecology
SDG: Sustainable development goal
