# Supplementary material for: Inequality of obstetric and gynaecological workforce distribution in China
Source: Int J Equity Health. 2018 Jan 5;17:3. doi: 10.1186/s12939-017-0716-6 (PMC5756396; doi:10.1186/s12939-017-0716-6)
Supplement: Additional file 1: — Table S1. The demographic distribution of OB/GYN workforce in the sampled districts/cities; Table S2. The geographic distribution of OB/GYN workforce in the sampled districts/cities. (DOCX 27 kb) [file 12939_2017_716_MOESM1_ESM.docx]

**Inequality of Obstetric and Gynaecological Workforce Distribution in China**

**Appendix Table S1.** The demographic distribution of OB/GYN workforce in the sampled districts/cities

| Region | Province | Sampled district/city | Number of OB/GYN doctors | | | | Number of OB/GYN nurses | | | | Number of midwives | | | | Number of OB/GYN workforce | | | |
| --- | --- | --- | --- | --- | --- | --- | --- | --- | --- | --- | --- | --- | --- | --- | --- | --- | --- | --- |
|  |  |  | /10,000 population | /10,000 women≥ 15 years | /10,000 women aged 15-49 years | /1,000 live births | /10,000 population | /10,000 women≥ 15 years | /10,000 women aged 15-49 years | /1,000 live births | /10,000 population | /10,000 women≥ 15 years | /10,000 women aged 15-49 years | /1,000 live births | /10,000 population | /10,000 women≥ 15 years | /10,000 women aged 15-49 years | /1,000 live births |
| East | Beijing | Xicheng | 4.66 | 10.12 | 16.07 | 72.33 | 4.86 | 10.58 | 16.79 | 75.56 | 1.50 | 3.26 | 5.17 | 23.27 | 11.02 | 23.96 | 38.03 | 171.16 |
|  | Beijing | Haidian | 0.98 | 2.20 | 2.91 | 19.04 | 1.20 | 2.69 | 3.56 | 23.30 | 0.51 | 1.14 | 1.51 | 9.87 | 2.69 | 6.02 | 7.98 | 52.21 |
|  | Beijing | Shunyi | 1.84 | 4.21 | 6.01 | 28.65 | 1.21 | 2.77 | 3.96 | 18.86 | 0.35 | 0.81 | 1.16 | 5.52 | 3.40 | 7.79 | 11.12 | 53.03 |
|  | Beijing | Pinggu | 3.01 | 6.86 | 10.99 | 42.96 | 2.57 | 5.87 | 9.40 | 36.77 | 0.43 | 0.99 | 1.58 | 6.19 | 6.01 | 13.71 | 21.97 | 85.91 |
|  | Tianjin | Hexi | 1.17 | 2.47 | 4.10 | 23.52 | 0.92 | 1.94 | 3.21 | 18.45 | 0.41 | 0.87 | 1.45 | 8.30 | 2.50 | 5.28 | 8.76 | 50.27 |
|  | Tianjin | Dongli | 1.80 | 4.62 | 6.46 | 27.01 | 1.54 | 3.94 | 5.51 | 23.01 | 0.23 | 0.60 | 0.84 | 3.50 | 3.57 | 9.16 | 12.81 | 53.51 |
|  | Tianjin | Wuqing | 1.18 | 2.91 | 4.37 | 16.40 | 0.84 | 2.08 | 3.12 | 11.71 | 0.24 | 0.60 | 0.90 | 3.37 | 2.26 | 5.59 | 8.38 | 31.47 |
|  | Tianjin | Jinghai | 1.41 | 3.52 | 5.22 | 12.81 | 1.22 | 3.06 | 4.53 | 11.12 | 0.17 | 0.43 | 0.63 | 1.55 | 2.80 | 7.01 | 10.38 | 25.48 |
|  | Hebei | Baoding | 2.20 | 5.30 | 7.73 | 17.00 | 2.05 | 4.95 | 7.22 | 15.88 | 1.14 | 2.75 | 4.01 | 8.81 | 5.39 | 12.99 | 18.97 | 41.70 |
|  | Liaoning | Yingkou | 1.35 | 3.12 | 4.85 | 21.75 | 1.28 | 2.96 | 4.60 | 20.63 | 0.49 | 1.13 | 1.75 | 7.87 | 3.13 | 7.21 | 11.21 | 50.24 |
|  | Shanghai | Yangpu | 1.84 | 3.98 | 7.15 | 40.36 | 3.24 | 6.99 | 12.56 | 70.88 | 0.55 | 1.18 | 2.13 | 12.01 | 5.63 | 12.15 | 21.84 | 123.25 |
|  | Shanghai | Minhang | 0.78 | 1.78 | 2.46 | 12.03 | 0.82 | 1.88 | 2.60 | 12.73 | 0.26 | 0.59 | 0.82 | 4.01 | 1.86 | 4.25 | 5.88 | 28.77 |
|  | Shanghai | Jiading | 0.84 | 1.98 | 2.69 | 17.28 | 0.99 | 2.32 | 3.14 | 20.20 | 0.36 | 0.85 | 1.15 | 7.38 | 2.19 | 5.15 | 6.98 | 44.87 |
|  | Shanghai | Chongming | 2.22 | 4.93 | 10.09 | 46.69 | 2.64 | 5.87 | 12.03 | 55.67 | 1.12 | 2.50 | 5.11 | 23.65 | 5.98 | 13.30 | 27.24 | 126.01 |
|  | Jiangsu | Huaian | 1.09 | 2.56 | 3.87 | 9.91 | 0.84 | 1.96 | 2.96 | 7.59 | 0.50 | 1.17 | 1.77 | 4.54 | 2.43 | 5.68 | 8.60 | 22.04 |
|  | Zhejiang | Jinhua | 1.98 | 4.74 | 6.73 | 22.89 | 0.96 | 2.29 | 3.26 | 11.07 | 0.98 | 2.35 | 3.34 | 11.35 | 3.92 | 9.39 | 13.33 | 45.32 |
|  | Fujian | Sanming | 1.36 | 3.36 | 4.82 | 11.12 | 0.84 | 2.07 | 2.97 | 6.85 | 1.92 | 4.75 | 6.80 | 15.69 | 4.12 | 10.18 | 14.59 | 33.65 |
|  | Shandong | Jining | 1.62 | 3.91 | 5.77 | 13.51 | 1.70 | 4.10 | 6.05 | 14.16 | 0.63 | 1.52 | 2.25 | 5.26 | 3.96 | 9.54 | 14.07 | 32.92 |
|  | Guangdong | Jiangmen | 2.61 | 6.19 | 8.85 | 28.99 | 2.94 | 6.97 | 9.97 | 32.65 | 0.83 | 1.96 | 2.80 | 9.17 | 6.38 | 15.11 | 21.61 | 70.81 |
|  | Guangdong | Chaozhou | 2.23 | 5.49 | 7.98 | 19.74 | 1.97 | 4.85 | 7.05 | 17.44 | 0.87 | 2.14 | 3.11 | 7.71 | 5.06 | 12.49 | 18.14 | 44.89 |
| Central | Shanxi | Jinzhong | 1.64 | 4.13 | 5.96 | 16.36 | 1.32 | 3.33 | 4.81 | 13.19 | 0.79 | 2.00 | 2.89 | 7.92 | 3.76 | 9.45 | 13.66 | 37.47 |
|  | Jilin | Baishan | 1.41 | 3.24 | 5.01 | 25.94 | 1.39 | 3.19 | 4.93 | 25.51 | 0.04 | 0.09 | 0.14 | 0.71 | 2.84 | 6.52 | 10.07 | 52.15 |
|  | Heilongjiang | Qitaihe | 1.42 | 3.35 | 4.81 | 20.14 | 0.95 | 2.23 | 3.19 | 13.37 | 0.47 | 1.10 | 1.58 | 6.61 | 2.84 | 6.68 | 9.58 | 40.12 |
|  | Anhui | Anqing | 1.36 | 3.24 | 4.69 | 14.03 | 1.29 | 3.08 | 4.46 | 13.35 | 0.52 | 1.23 | 1.78 | 5.33 | 3.16 | 7.55 | 10.93 | 32.71 |
|  | Jiangxi | Ganzhou | 1.64 | 4.28 | 6.05 | 12.38 | 1.57 | 4.10 | 5.79 | 11.84 | 0.76 | 1.98 | 2.80 | 5.71 | 3.97 | 10.36 | 14.64 | 29.93 |
|  | Henan | Louyang | 1.89 | 4.73 | 6.83 | 17.68 | 1.61 | 4.01 | 5.80 | 15.00 | 0.61 | 1.51 | 2.18 | 5.66 | 4.11 | 10.25 | 14.81 | 38.33 |
|  | Henan | Xinyang | 2.38 | 6.03 | 8.92 | 19.58 | 1.67 | 4.23 | 6.25 | 13.73 | 0.71 | 1.80 | 2.66 | 5.85 | 4.77 | 12.05 | 17.83 | 39.16 |
|  | Hubei | Huanggang | 1.96 | 4.71 | 6.99 | 17.88 | 2.09 | 5.02 | 7.45 | 19.05 | 0.79 | 1.90 | 2.82 | 7.20 | 4.84 | 11.63 | 17.26 | 44.13 |
|  | Hunan | Yiyang | 1.45 | 3.44 | 5.07 | 12.59 | 1.75 | 4.16 | 6.13 | 15.21 | 0.31 | 0.73 | 1.08 | 2.68 | 3.51 | 8.34 | 12.28 | 30.48 |
| West | Inner Mongolia | Bayannaoer | 1.49 | 3.68 | 5.23 | 20.34 | 1.37 | 3.37 | 4.79 | 18.63 | 0.54 | 1.33 | 1.89 | 7.35 | 3.40 | 8.37 | 11.91 | 46.32 |
|  | Guangxi | Baise | 2.88 | 7.44 | 10.97 | 20.75 | 1.99 | 5.14 | 7.58 | 14.32 | 1.30 | 3.35 | 4.94 | 9.33 | 6.16 | 15.92 | 23.49 | 44.40 |
|  | Chongqing | Nanan | 3.69 | 8.35 | 12.00 | 51.95 | 3.55 | 8.05 | 11.58 | 50.09 | 1.28 | 2.89 | 4.16 | 18.00 | 8.52 | 19.30 | 27.74 | 120.04 |
|  | Chongqing | Yongchuan | 2.23 | 5.39 | 8.34 | 20.72 | 1.60 | 3.87 | 6.00 | 14.91 | 0.29 | 0.71 | 1.10 | 2.73 | 4.12 | 9.97 | 15.44 | 38.36 |
|  | Chongqing | Qijiang | 0.99 | 2.38 | 3.88 | 10.45 | 1.45 | 3.50 | 5.70 | 15.34 | 0.41 | 0.99 | 1.62 | 4.36 | 2.85 | 6.87 | 11.21 | 30.15 |
|  | Chongqing | Chengkou | 2.44 | 6.45 | 9.94 | 17.18 | 1.30 | 3.43 | 5.28 | 9.14 | 0.36 | 0.96 | 1.48 | 2.56 | 4.09 | 10.85 | 16.70 | 28.88 |
|  | Sichuan | Mianyang | 2.69 | 6.30 | 9.76 | 31.17 | 2.30 | 5.38 | 8.33 | 26.60 | 0.57 | 1.32 | 2.05 | 6.55 | 5.55 | 13.01 | 20.15 | 64.33 |
|  | Sichuan | Ziyang | 2.51 | 6.13 | 10.40 | 25.51 | 2.15 | 5.26 | 8.93 | 21.91 | 0.46 | 1.13 | 1.92 | 4.72 | 5.12 | 12.53 | 21.25 | 52.14 |
|  | Guizhou | Qiannan | 1.34 | 3.55 | 5.34 | 9.36 | 1.31 | 3.46 | 5.20 | 9.13 | 0.67 | 1.78 | 2.67 | 4.68 | 3.32 | 8.80 | 13.20 | 23.17 |
|  | Yunnan | Xishuangbanna | 1.21 | 3.08 | 4.05 | 10.42 | 0.87 | 2.22 | 2.93 | 7.53 | 0.42 | 1.08 | 1.42 | 3.65 | 2.51 | 6.38 | 8.41 | 21.61 |
|  | Shaanxi | Xi'an | 2.44 | 5.71 | 8.12 | 30.05 | 2.58 | 6.04 | 8.59 | 31.76 | 1.07 | 2.49 | 3.55 | 13.12 | 6.10 | 14.24 | 20.26 | 74.94 |
|  | Gansu | Jinchang | 1.62 | 3.98 | 5.54 | 19.39 | 1.55 | 3.82 | 5.31 | 18.61 | 0.34 | 0.85 | 1.18 | 4.14 | 3.51 | 8.65 | 12.03 | 42.14 |
|  | Qinghai | Xining | 8.46 | 20.93 | 28.25 | 84.17 | 2.17 | 5.38 | 7.26 | 21.63 | 0.69 | 1.70 | 2.30 | 6.85 | 11.32 | 28.02 | 37.81 | 112.64 |
|  | Ningxia | Zhongwei | 2.88 | 7.68 | 10.06 | 22.76 | 2.80 | 7.48 | 9.80 | 22.18 | 1.02 | 2.72 | 3.56 | 8.05 | 6.70 | 17.87 | 23.43 | 52.99 |
|  | Xinjiang | Yili | 2.17 | 5.64 | 7.28 | 13.04 | 2.34 | 6.09 | 7.87 | 14.08 | 1.19 | 3.10 | 4.01 | 7.17 | 5.70 | 14.83 | 19.16 | 34.29 |

**Appendix Table S2.** The geographic distribution of OB/GYN workforce in the sampled districts/cities

| Region | Province | Sampled district/city | Number of OB/GYN doctors/square kilometre | Number of OB/GYN nurses/square kilometre | Number of midwives/square kilometre | Number of OB/GYN workforce/square kilometre |
| --- | --- | --- | --- | --- | --- | --- |
| East | Beijing | Xicheng | 6.193 | 6.469 | 1.992 | 14.655 |
|  | Beijing | Haidian | 0.747 | 0.915 | 0.388 | 2.050 |
|  | Beijing | Shunyi | 0.158 | 0.104 | 0.030 | 0.292 |
|  | Beijing | Pinggu | 0.116 | 0.100 | 0.017 | 0.233 |
|  | Tianjin | Hexi | 2.757 | 2.162 | 0.973 | 5.892 |
|  | Tianjin | Dongli | 0.235 | 0.200 | 0.030 | 0.465 |
|  | Tianjin | Wuqing | 0.071 | 0.051 | 0.015 | 0.137 |
|  | Tianjin | Jinghai | 0.062 | 0.054 | 0.007 | 0.123 |
|  | Hebei | Baoding | 0.111 | 0.104 | 0.057 | 0.272 |
|  | Liaoning | Yingkou | 0.061 | 0.058 | 0.022 | 0.140 |
|  | Shanghai | Yangpu | 3.993 | 7.012 | 1.188 | 12.193 |
|  | Shanghai | Minhang | 0.509 | 0.538 | 0.170 | 1.216 |
|  | Shanghai | Jiading | 0.267 | 0.312 | 0.114 | 0.694 |
|  | Shanghai | Chongming | 0.137 | 0.163 | 0.069 | 0.369 |
|  | Jiangsu | Huaian | 0.052 | 0.040 | 0.024 | 0.116 |
|  | Zhejiang | Jinhua | 0.097 | 0.047 | 0.048 | 0.192 |
|  | Fujian | Sanming | 0.015 | 0.009 | 0.021 | 0.045 |
|  | Shandong | Jining | 0.117 | 0.123 | 0.046 | 0.286 |
|  | Guangdong | Jiangmen | 0.122 | 0.138 | 0.039 | 0.299 |
|  | Guangdong | Chaozhou | 0.161 | 0.143 | 0.063 | 0.367 |
| Central | Shanxi | Jinzhong | 0.032 | 0.026 | 0.016 | 0.074 |
|  | Jilin | Baishan | 0.010 | 0.010 | 0.000 | 0.021 |
|  | Heilongjiang | Qitaihe | 0.021 | 0.014 | 0.007 | 0.042 |
|  | Anhui | Anqing | 0.053 | 0.050 | 0.020 | 0.124 |
|  | Jiangxi | Ganzhou | 0.035 | 0.033 | 0.016 | 0.084 |
|  | Henan | Louyang | 0.081 | 0.069 | 0.026 | 0.177 |
|  | Henan | Xinyang | 0.077 | 0.054 | 0.023 | 0.154 |
|  | Hubei | Huanggang | 0.069 | 0.074 | 0.028 | 0.171 |
|  | Hunan | Yiyang | 0.051 | 0.062 | 0.011 | 0.124 |
| West | Inner Mongolia | Bayannaoer | 0.004 | 0.004 | 0.001 | 0.009 |
|  | Guangxi | Baise | 0.028 | 0.019 | 0.012 | 0.059 |
|  | Chongqing | Nanan | 1.064 | 1.026 | 0.369 | 2.459 |
|  | Chongqing | Yongchuan | 0.145 | 0.104 | 0.019 | 0.268 |
|  | Chongqing | Qijiang | 0.029 | 0.042 | 0.012 | 0.083 |
|  | Chongqing | Chengkou | 0.014 | 0.008 | 0.002 | 0.024 |
|  | Sichuan | Mianyang | 0.061 | 0.052 | 0.013 | 0.126 |
|  | Sichuan | Ziyang | 0.115 | 0.099 | 0.021 | 0.236 |
|  | Guizhou | Qiannan | 0.017 | 0.016 | 0.008 | 0.041 |
|  | Yunnan | Xishuangbanna | 0.007 | 0.005 | 0.002 | 0.015 |
|  | Shaanxi | Xi'an | 0.205 | 0.216 | 0.089 | 0.511 |
|  | Gansu | Jinchang | 0.008 | 0.008 | 0.002 | 0.017 |
|  | Qinghai | Xining | 0.244 | 0.063 | 0.020 | 0.327 |
|  | Ningxia | Zhongwei | 0.021 | 0.021 | 0.007 | 0.049 |
|  | Xinjiang | Yili | 0.002 | 0.002 | 0.001 | 0.005 |
